# Supplementary material for: Toward Oral Supplementation of Diamine Oxidase for the Treatment of Histamine Intolerance
Source: Nutrients. 2022 Jun 24;14(13):2621. doi: 10.3390/nu14132621 (PMC9268349; doi:10.3390/nu14132621)
Supplement: Supplementary file 1 [file nutrients-14-02621-s001.zip › nutrients-1731933-supplementary.pdf]

Toward Oral Supplementation of Diamine Oxidase for the Treatment of Histamine Intolerance

Lucas Kettner, Ines Seitl, Lutz Fischer*

Department of Biotechnology and Enzyme Science,

Institute of Food Science and Biotechnology,

University of Hohenheim

Garbenstr. 25, 70599 Stuttgart, Germany

* Corresponding author: Lutz Fischer

E-mail address: Lutz.Fischer@uni-hohenheim.de

Tel.: +49 711 459-22311

Supplementary Materials

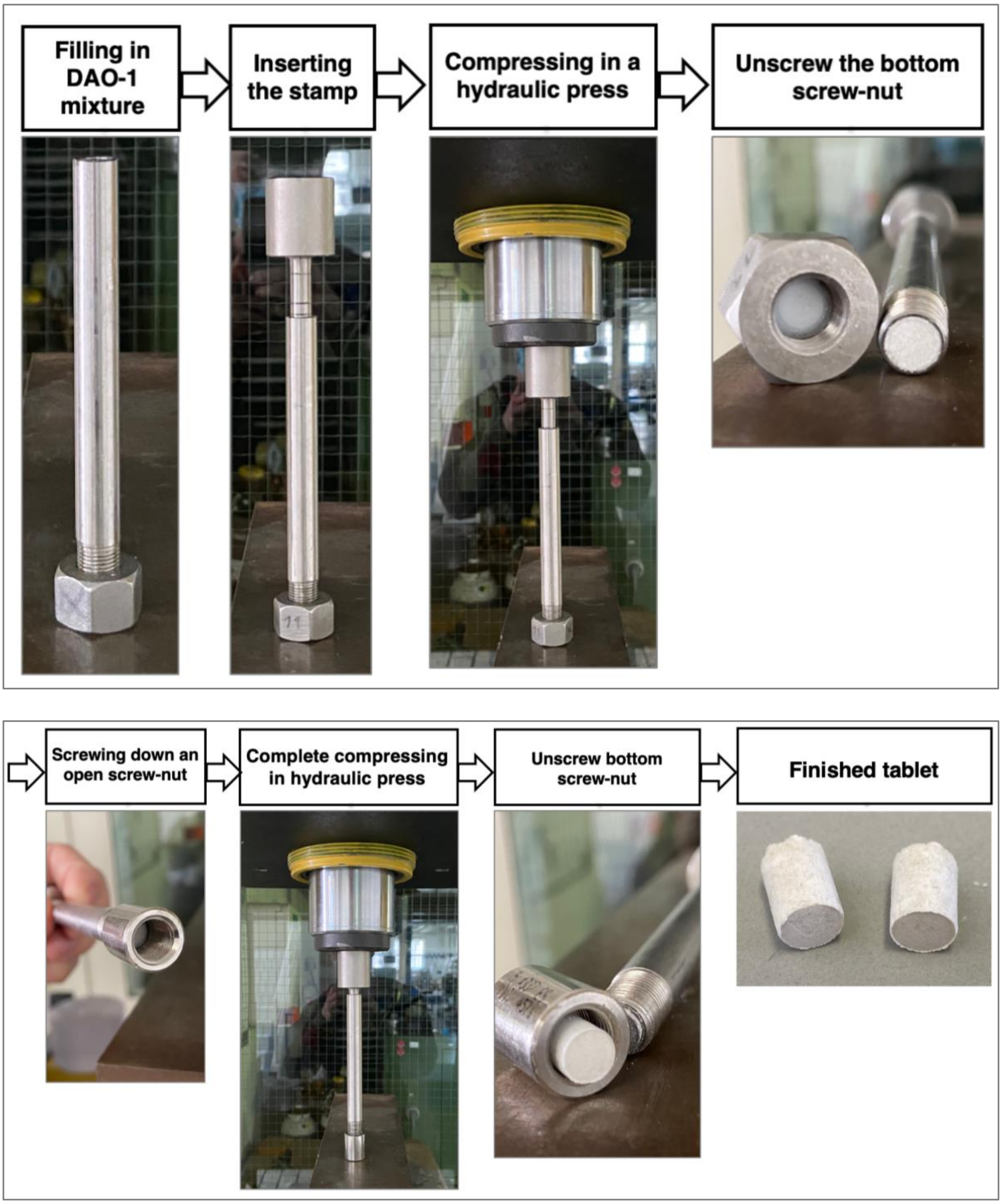

Figure S1. Preparation of DAO-1 tablets using a self-built tablet press.

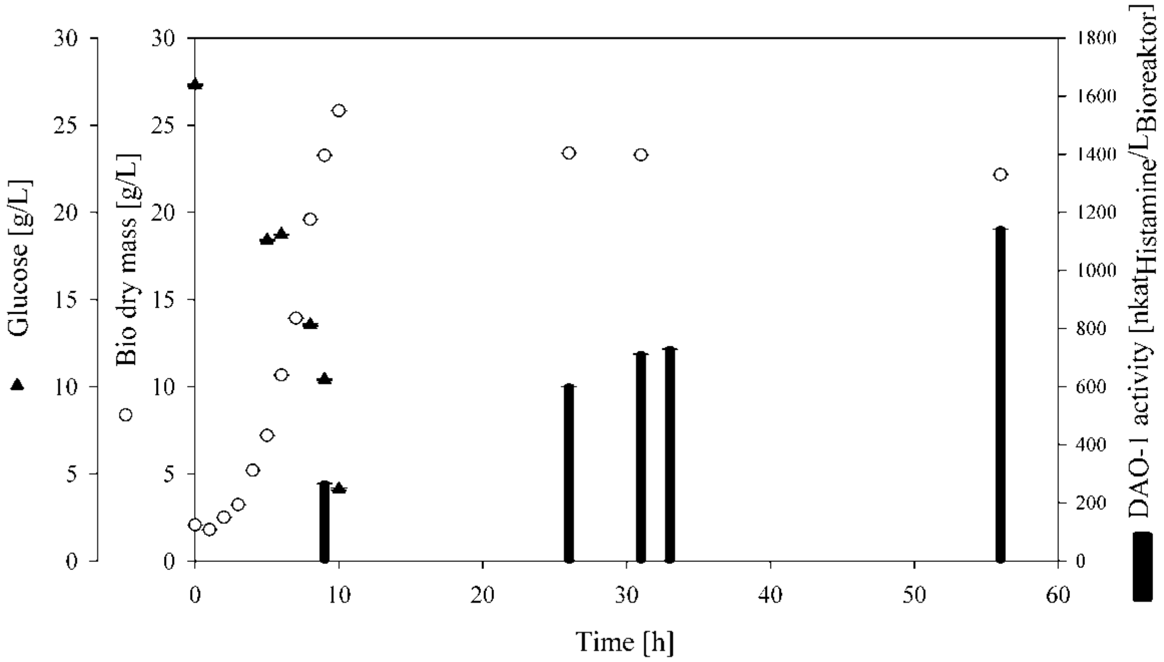

Figure S2. Bioreactor cultivation of *Yarrowia lipolytica_axp_dao*-1 in YPD$_{Ura}$ media; working volume 5 l at 28 °C (pH 6.5). *Y. lipolytica* cells were disrupted for DAO-1 activity determinations using the TissueLyser II (Qiagen, Düsseldorf, Germany) with glass beads (0.75 mm in diameter). DAO-1 activity was determined under standard assay conditions using the DA-67 assay.

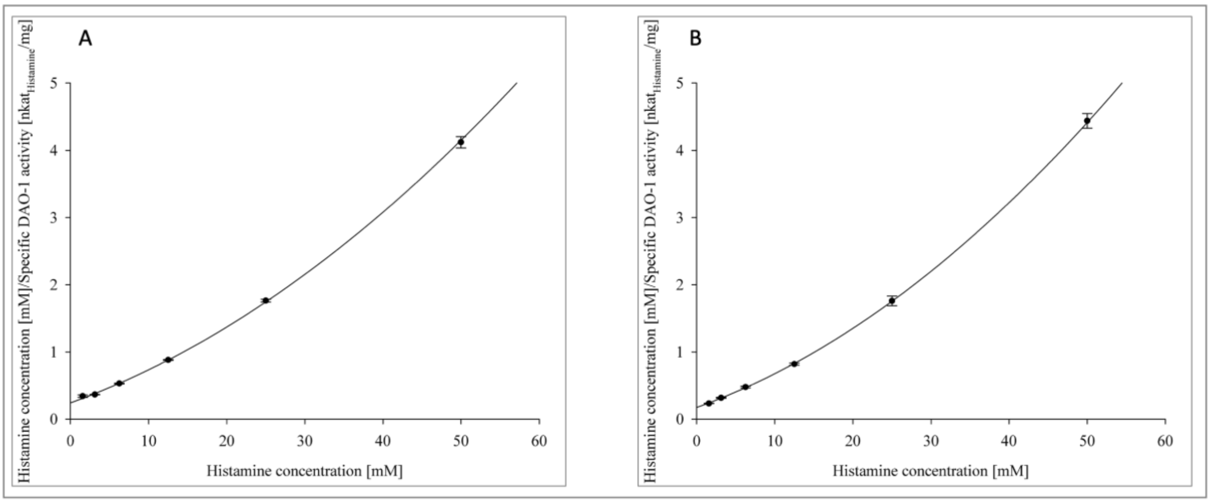

Figure S3. Michaelis-Menten kinetics of DAO-1 in a pancreatin-free and pancreatin-treated food-matrix simulated intestinal fluid (SIF 3) at 37 °C. Linearization according to Hanes-Woolf. Histamine concentration ranging from 1.56 to 50 mM.

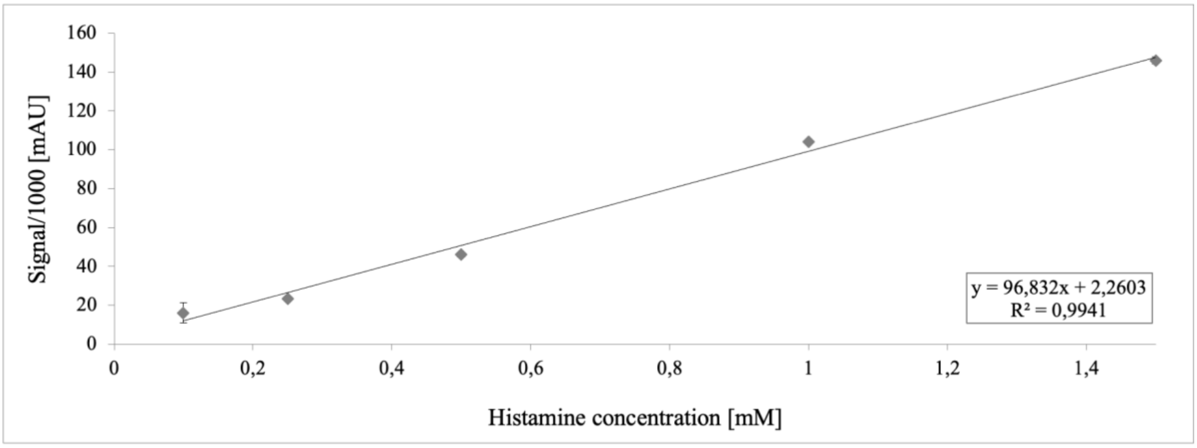

Figure S4. Exemplary histamine calibration for the quantification of histamine in the food-matrix SIF bioconversion samples (90 min) on the AquaC18 column (150 x 4.6 mm; 200 Å) from Phenomenex (Platinblue UHPLC system from Knauer (Berlin, Germany)). Detection at 210 nm.
